# Autotoxicity effect of water extracts from rhizosphere soil of *Elymus sibiricus* in different planting years on seed germination, physiological characteristics and phytohormones of seedlings

**DOI:** 10.7717/peerj.13768

**Published:** 2022-07-28

**Authors:** Hang Yang, Jinglong Su, Juan Qi

**Affiliations:** 1 College of Grassland Science, Gansu Agricultural University, Lanzhou, China; 2 Centers for Grazing Land Ecosystem Sustainability, Gansu Agricultural University, Lanzhou, China; 3 Pratacultural Engineering Laboratory of Gansu Province, Sino-U.S, Lanzhou, China

**Keywords:** *E. sibiricus*, Planting years, Rhizosphere soil extracts, Autotoxicity

## Abstract

*Elymus sibiricus* is a highly valuable perennial forage that is widely planted in the Qinghai-Tibet Plateau (QTP) region. However, *E. sibiricus* artificial grasslands have a short utilization lifespan, and reach the highest yield in the 2nd and 3rd year of plantation, then rapidly drop its productivity. We hypothesized that autotoxicity is one of the mechanisms for the reduction of the productivity. To test this hypothesis, we prepared the water extract from rhizosphere soils of *E. sibiricus* planted for 3, 4, 5, and 8 years and examined the effects of the extract concentrations at 0.05, 0.1, 0.2, and 0.5 g/mL on seed germination, seedling growth, physiological characteristics and phytohormones in the aboveground and roots of *E. sibiricus*. The results showed that the soil extract concentration, planting years, and their interaction had significant influences on the most of these indices. The soil extract inhibited the seed germination and growth of seedlings, and the inhibitory effects appeared to be stronger at the 0.5 g/mL rhizosphere soil extract for 5 and 8 years. The superoxide dismultase and peroxide activities, the free proline concentration, soluble sugar concentration were altered. The malondialdehyde concentration was, in general, increased, especially in 8 years soil extract. The indole acetic acid and gibberellic acids concentrations were lowered, while the abscisic acid concentration varied. These changes were depending on the extract concentration and the years of planting, without clear patterns in some of them in response to the extract concentration and planting years. In summary, autotoxicity can be a contributor to the retardation of the growth and development of artificial *E. sibiricus* grasslands. The inhibitory effects could be attribute to impaired antioxidant capacity and disturbance of osmortic-regulatory substances and plant hormones, and are more profound on the root than on the aboveground part of the seedlings.

## Introduction

*Elymus sibiricus* belongs to *Elymus* genus and is indigenous perennial species in Northern Asia and widely spreads in the Qinghai-Tibet Plateau (QTP) region. It presents in natural grasslands and is also used for artificial grasslands due to its strong adaptability, highly nutritional quality, and good palatability for grazing animals (Yan et al., 2007). Some studies reported that only a few gramineous species can adapt to the local environment in the Alpine meadow region (Zhang et al., 2014; Zhang, Ma & Du, 2017), among them *E. sibiricus* is one that can be easy to establish. Under the local environment conditions, *E. sibiricus* has the higher yield in the 2nd and 3rd year after the planting for artificial grasslands, and the yield begins declining from the 4th year, which raises concerns on economic benefits and ecological sustainability (Dong, Ma & Zhao, 2007). It seems obvious that with the increase of planting years for *E. sibiricus*, there develops imbalance between nutrients and microorganisms in soils, which will impact plant growth (Liu et al., 2020).

Allelopathy is an effect of a donor plant on a recipient plant by releasing chemical compounds into the local environment (Rice, 1984), and autotoxicity occurs when the recipient plant is conspecific as the donor plant (Kato-Noguchi, Nakamura & Okuda, 2018). This phenomenon, also known as autoallelopathy or simply phytotoxins, occurs for a great number of plants in the agricultural ecosystem (Sun & He, 2019). In autotoxicity, plants can release allelochemicals via decomposition, volatilization, leaching, and root exudation (El Mehdawi, Quinn & Pilon-Smits, 2011), and the chemical nature of the compounds is the secondary metabolites include simple organic acids, straight-chain alcohols, aldehydes or ketones, lactones, unsaturated fatty acids, naphthoquinones, quinone complex, simple phenols, tannins, terpenoids, amino acids, polypeptides, alkaloids, glucosinolates, purines, and nucleotides (Gross & Parthier, 1994; Rice, 1995). Some water-soluble compounds can also be released into the local environment through rainfall, leaching, *etc*. (Morshedloo et al., 2017). The accumulation of allelochemicals in soils and plants can reduce the seed germination rate, crop yield and quality (Hao et al., 2007; Zhang et al., 2021b), and increase the development of soil-borne diseases (Huang et al., 2013; Wu et al., 2015), triggering a series of physiological variations in affected plants (Yang et al., 2017; Wang, Deng & Yu, 2019) by allelochemical stress. Under allelochemical stress, plants produce a large amount of reactive oxygen species (ROS) which can cause peroxidation of lipids in the membrane and cell death (Li et al., 2016). It has been found that a treatment of alfalfa seeds using water extract of alfalfa leaves could scavenge ROS by changing the activities of superoxide dismutase (SOD), peroxidase (POD) and catalase (CAT) in seedlings (Zhang et al., 2021b). Excessive ROS can also change osmotic pressure, resulting in the destruction of cell structure and loss of functions (Zhang et al., 2020a; Zhang et al., 2020b). Under oxidative stress, the contents of malondialdehyde (MDA), soluble sugar and soluble protein in plants increase (Davey et al., 2005; Zhang et al., 2021b). In addition, allelochemicals may affect plant hormone profiles (Wu, Cao & Zhang, 2008; Ren et al., 2018), and a study found the methanol extract of *Lepidium draba* increased the abscisic acid (ABA) level and decreased the gibberellic acid (GA) level in corn (Kaya et al., 2015). Decomposed liquid of corn stalk can disturb the metabolism of endogenous hormones in its seedlings, and the accumulation of indole acetic acid (IAA), GA and zeatin riboside (ZR) were increased, so plant growth was inhibited (Li et al., 2015). Autotoxicity is generally considered to be the main cause of replanting problems and has been documented in various plants (Ren et al., 2015), and effect of autotoxicity seems to relate to the allelochemical concentration, as one study showed that seed germination and the initial growth biomass were inversely correlated with the concentration of the water extract of leaves and roots of plants (Favaretto, Scheffer-Basso & Perez, 2017). Deng et al. (2017) discovered that the root exudates of tobacco inhibited its germination and growth of tobacco, and found the dioctyl phthalate and diisooctyl phthalate were the main autotoxic substances exuded by roots. Zhang et al. (2020a) study found that autotoxicity decreased the starch degradation ability of melon seeds during germination, increased lipid peroxidation of cell membrane and abnormal antioxidant enzyme activity. *Picea schrenkiana* autotoxic substance 3,4-dihydroxy-acetophenone increased the content of IAA and GA in its seedlings under low concentration treatment (0.1–0.25 mM), while high concentration (0.5–1.0 mM) significantly decreased the content of IAA and GA and increased the concentration of ABA (Yang et al., 2017). Therefore, we hypothesized that autotoxicity could be one of the mechanisms responsible for the degeneration of *E. sibiricus* after two years of planting.

Plant roots during the growth can release allelochemicals into rhizosphere soils, and it has been found the water extract of rhizosphere soils had an inhibitory effect on plants growth (Kato-Noguchi, 2020). Up to now, the research on plant autotoxicity focuses mainly on food crops (Yu et al., 2020), and little research is on gramineous forages. In the present study, we used the water extract of rhizosphere soils from *E. sibiricus* planted for 3, 4, 5, and 8 years and tested the effects of different concentration on seed germination, physiological characteristics and phytohormones of seedlings. The aim was to identify the roles of autotoxicity in the retardation of growth and development of *E. sibiricus*.

## Materials and Methods

### Experimental design

This experiment examined the effects of four concentrations of rhizosphere soils of *E. sibiricus* planted already for 3, 4, 5, and 8 years on seed germination, physiological characteristics and phytohormones of seedlings of *E. sibiricus.* Plus a water control to the soil extracts, it was a typical 5 × 4 factorial design.

In September 2019, the evenly growing roots of *E. sibiricus* were excavated from the *E. sibiricus* artificial grassland that had been planted for 3, 4, 5, and 8 years. There was no decline in the 3 years grassland, a clear decline in the 5 and 8 years grassland, and the 4 years grassland could not be clearly distinguished from the degraded state. The field management during the growth period of each year was the same, weeds were manually controlled and no fertilizer or irrigation was carried out. The grassland is located in the National Grass Variety Regional Test Center (100°85′E and N36°45′E) in Qinghai Province, China. *E. sibiricus* was in the wax maturity period. The rhizosphere soils attached to the excavated roots were shaken off, collected into a sealed bag, and stored at −20 °C. *E. sibiricus* seeds were collected from the National Grass Variety Regional Test Center in October 2018.

The collected rhizosphere soil was air dried and grinded to pass a 0.25 mm sieve. Then 5, 10, 20 and 50 g were weighted into glass bottles, and 100 mL of distilled water was added, heated up to 30 °C under ultrasonic waves for 30 min. The bottle was kept at 25 °C for 24 h with consistently shaking at 150 rpm. The supernatant was filtered through 0.45 µm filter paper and the filtrate stored at 4 °C until the use. Therefore, the extract concentrations were 0.05, 0.1, 0.2, and 0.5 g of equivalent rhizosphere soil per mL of the solution, and the total number of the extract was 16 across 3, 4, 5, and 8 years.

### Seed germination test

For each of the soil extract, 30 seeds of *E. sibiricus* were selected and placed on two layers of filter paper on a 9 cm diameter petri dish, five mL of the soil extract were added. The 5 mL distilled water was used as the control. The test was triplicated for each extract. Next, petri dishes were placed in an incubator at 20 °C with photoperiod of 12/12 h dark/light cycles (HGZ-H, Shanghai Hengyue Medical Instrument Co., Ltd, China). Radicle extend of 2 mm from the seed coat was considered as the germination. The number of germinated seeds was recorded daily. The germination force on the 5 days (GF), and the percentage of seed germination up to 12 days (GP) were calculated. On the day, 4 seedlings for each treatment were randomly selected for measurements of shoot length (SL) and root length (RL) with a ruler.

### Seedling growth measurement

Seedlings were cultivated using a sand culture method. Fine sands were filled into a disposable plastic cup with 16 cm depth, 11 cm top diameter and 10 cm bottom diameter. Then 50 *E. sibiricus* seeds were dispensed evenly on the surface of sands, and covered with 1 cm of sands. The cups were placed in an incubator and cultivated in a condition as followings: cycles of 25 °C with 14 h light and 20 °C with 10 h darkness, luminous flux density 400 µmol/m^2^ s^1^, and relative humidity 60%. Distilled water was irrigated daily before seedling emergence. After the emergence, each cup was irrigated with 10 mL of the soil extract or distilled water (the control) and 30 mL of the 1/2 Hoagland nutrient solution every other day. There were triplicated cups for each treatment. Thirty days after the seeds were planted and grown, seedlings were carefully taken out of the sands, separated into the aboveground part and roots, respectively, and stored at −80 °C until further determinations.

### Index measurement

#### Determinations of GF, GP and allelopathic effect response index (RI)

GF, GP and the allelopathic effect response index (RI) were calculated according to the equation as followings (Williamson & Richardson, 1988; Jespersen, Yu & Huang, 2017):

\begin{eqnarray*}\mathrm{GF}& & ={\mathrm{N}}_{5}/{\mathrm{N}}_{\mathrm{total}}\times 100\text{%} \end{eqnarray*}

\begin{eqnarray*}\mathrm{GP}& & ={\mathrm{N}}_{12}/{\mathrm{N}}_{\mathrm{total}}\times 100\text{%} \end{eqnarray*}

\begin{eqnarray*}\mathrm{RI}& & =1-\mathrm{C/T}~(\mathrm{T}\geq \mathrm{C})~\mathrm{or}~\mathrm{T/C}-1~(\mathrm{T}\lt \mathrm{C}) \end{eqnarray*}

where N_5_ is the number of germinated seeds on day 5, N_12_ is the number of germinated seeds on day 12, N_total_ is the total number of seeds for the test (30 in this experiment), T is the treatment value, C is the corresponding value for the Control. Positive values of RI show a stimulatory effect and negative ones indicate inhibitory activity of the aqueous extract.

### Seedlings’ physiological parameters assays

For the aboveground part and roots of seedlings, respectively, the SOD activity was determined using the nitrogen blue tetrazole method (Liu et al., 2009). The POD activity was determined using the guaiacol method (Zhang et al., 2007). The MDA concentration was determined using the thiobarbituric acid method (Draper & Hadley, 1990). Free proline concentration was assayed using the acid-ninhydrin method (Bates, Waldren & Teare, 1973). The soluble protein was determined using the bovine serum albumin (Bradford, 1976). The soluble sugar was assayed by an anthrone colorimetric method (Buysse & Merckx, 1993).

### Determinations of phytohormones

To determine phytohormones concentration in the aboveground part and roots of *E. sibiricus* seedlings, 2.5 g fresh sample was weighed out in a mortar and grinted in liquid nitrogen. Sample was transferred into a 50 mL centrifuge tube, 20 mL of 80% methanol were added, kept at 4 °C for 16 h, and then centrifuged at 10,000 g at 4 °C for 15 min. The supernatant was transferred into a 50 mL centrifuge tube. The residue was then extracted with 15 mL of 80% methanol and ultrasonicated for 30 min, centrifuged at 10,000 g at 4 °C for 15 min. The supernatant was transferred into the 50 mL centrifuge tube, so two extractions were pooled. The extract solution was evaporated in a rotary concentrator at 40 °C until the volume down to about 20 mL, then decolorization of the solution was performed twice by adding 15 mL of petroleum ether each time, mixed, and sucked out. The concentration process in the evaporator was continued until dry, then two mL of 80% methanol was added to form the test solution. The test solution was filtered through 0.22 µm organic filter membrane into a 2 mL sample vial for assays of IAA, GA, and ABA using a Quaternary Gradient ultra-Fast Liquid Chromatography (Waters ACQUITY Arc Bi, Waters Technologies Shanghai Limited, China) (Zhang et al., 2013). The assay conditions were set as: injection volume 10 µL, flow rate 1 mL/min, mobile phase consisting of methanol in 0.1% phosphoric acid (35:65), and column temperature at 30 °C.

### Statistical analysis

For this 5 (the soil extract concentrations) ×4 (years of planting) designed experiment, two-way analysis variance (ANOVA) model was used to analyze the data. The soil extract concentrations and years of planting are the factors, and their interaction was included in the model. The differences between the means were compared by Fisher’s protected least significant difference test (LSD), and statistically significant was declared as a *P* value ≤ 0.05. The data are presented as means and standard error of the means (SEM).

## Results

### Seed germination and seedling characteristics

The interactions between years of planting and the extract concentrations of rhizosphere soil had significant effects on the GF (*df* = 12; *P* < 0.001), GP (*df* = 12; *P* = 0.02), and SL (*df* = 12; *P* < 0.001), but not on RL (*df* = 12; *P* = 0.54). The GF (*df* = 3; *P* = 0.00) and SL (*df* = 3; *P* = 0.02) were significantly and affected by the years of planting but not GP (*df* = 12; *P* = 0.38) and RL (*df* = 12; *P* = 0.23), while the extract concentrations significantly (*df* = 4; *P* < 0.001) affected GP, SL, RL, and GF (Table 1). Compared with the control, in general, the concentrations of the soil extract had inhibitory effects on seed germination (GF, GP) and seedling growth (SL, RL), particularly at the highest concentration of 0.5 g/mL. The inhibitory effects varied depending on the years of planting, and appeared to be more profound on SL and RL by the soil extract from *E. sibiricus* planted for 5 and 8 years.

**Table 1:** Effects of water extract of rhizosphere soils of *E. sibiricus* in different planting years on germination force (GF), germination percentage (GP) of seeds, and shoot length (SL), root length (RL) of *E. sibiricus* of seedlings. Means with different superscript lowercase letters indicate significant differences between different concentrations of the same planting years, and upper case letters indicate significant differences between different planting years of the same concentration at *P* < 0.05 (LSD test).

	Concentrations of soil extract (g/mL)	Years of planting				Significance (*P* values)		
		3	4	5	8	Years	Concentrations	Y ×C
Germination force (%)	0	51.11 ± 2.94^Aa^	51.11 ± 2.94^Aa^	51.11 ± 2.94^Aa^	51.11 ± 2.94^Aa^	0.00	0.00	0.00
	0.05	48.89 ± 4.01^Aa^	42.22 ± 4.01^Aab^	47.78 ± 2.94^Aa^	38.89 ± 2.94^Ab^			
	0.1	32.22 ± 2.94^Bb^	36.67 ± 1.92^ABb^	43.33 ± 3.85^Aa^	35.56 ± 2.94^ABb^			
	0.2	43.33 ± 3.85^Aa^	42.22 ± 2.94^Aab^	32.22 ± 1.11^Bb^	35.56 ± 1.11^ABb^			
	0.5	30.00 ± 3.33^Bb^	37.78 ± 1.11^Ab^	30.00 ± 1.92^Bb^	33.33 ± 1.92^ABb^			
Germination percentage (%)	0	81.00 ± 1.00^Aa^	81.00 ± 1.00^Aa^	81.00 ± 1.00^Aa^	81.00 ± 1.00^Aa^	0.38	0.00	0.02
	0.05	68.00 ± 1.00^Ab^	62.33 ± 4.67^Ab^	65.67 ± 5.93^Ab^	62.33 ± 2.91^Ab^			
	0.1	55.67 ± 1.33^Bc^	63.00 ± 0.00^Ab^	61.00 ± 1.00^ABbc^	55.67 ± 2.96^Bc^			
	0.2	69.00 ± 2.00^Ab^	67.00 ± 0.00^Ab^	53.33 ± 2.03^Bcd^	53.00 ± 0.00^Bc^			
	0.5	52.33 ± 2.33^Ac^	53.33 ± 2.03^Ac^	45.67 ± 1.33^Bd^	50.00 ± 0.00^ABc^			
Shoot length(cm)	0	5.63 ± 0.09^Aa^	5.63 ± 0.09^Aa^	5.63 ± 0.09^Aa^	5.63 ± 0.09^Aa^	0.02	0.00	0.00
	0.05	5.58 ± 0.23^Aa^	4.80 ± 0.15^Bbc^	5.33 ± 0.14^Aa^	4.40 ± 0.07^Bd^			
	0.1	5.15 ± 0.12^Bab^	5.58 ± 0.05^Aa^	4.98 ± 0.05^BCb^	4.90 ± 0.07^Cc^			
	0.2	4.45 ± 0.13^Bc^	5.15 ± 0.18^Ab^	4.88 ± 0.10^Ab^	5.30 ± 0.12^Ab^			
	0.5	4.93 ± 0.16^Ab^	4.73 ± 0.13^ABc^	4.48 ± 0.13^Bc^	4.53 ± 0.06^ABd^			
Root length(cm)	0	5.80 ± 0.12^Aa^	5.80 ± 0.12^Aa^	5.80 ± 0.12^Aa^	5.80 ± 0.12^Aa^	0.23	0.00	0.54
	0.05	3.83 ± 0.20^Ab^	3.43 ± 0.13^Ab^	3.55 ± 0.23^Ab^	3.55 ± 0.17^Ab^			
	0.1	3.48 ± 0.16^Abc^	3.45 ± 0.19^Ab^	2.93 ± 0.17^Ac^	3.38 ± 0.38^Abc^			
	0.2	3.30 ± 0.04^Ac^	3.15 ± 0.12^ABb^	2.93 ± 0.08^Bc^	2.88 ± 0.13^Bcd^			
	0.5	2.75 ± 0.14^Ad^	2.98 ± 0.21^Ab^	2.93 ± 0.23^Ac^	2.68 ± 0.11^Ad^			

The extract concentrations had inhibitory effects on seed germination and seedling growth, the allelopathic response of GF, GP, and SL decreased with the increasing concentrations of the extract from *E. sibiricus* planted for 5 years, and the allelopathic response indexes of RL decreased with the increasing concentrations of the extract from *E. sibiricus* planted for 3 and 8 years (Table 2).

**Table 2:** Allelopathic response indexes of *E. sibiricus* to rhizosphere soil extract. Means with different superscript lowercase letters indicate significant differences between different concentrations of the same planting years, and upper case letters indicate significant differences between different planting years of the same concentration at *P* < 0.05 (LSD test).

Allelopathic response	Concentration of soil extract (g/mL)	Years of planting			
		3 years	4 years	5 years	8 years
Germination force	0.05	−0.05 ± 0.07^Aa^	−0.17 ± 0.08^Aa^	−0.07 ± 0.06^Aa^	−0.24 ± 0.06^Aa^
	0.1	−0.37 ± 0.06^Bbc^	−0.28 ± 0.04^ABa^	−0.15 ± 0.08^Aa^	−0.30 ± 0.06^ABa^
	0.2	−0.15 ± 0.08^Aab^	−0.17 ± 0.06^Aa^	−0.37 ± 0.02^Bb^	−0.30 ± 0.02^ABa^
	0.5	−0.41 ± 0.07^Bc^	−0.26 ± 0.02^Aa^	−0.41 ± 0.04^Bb^	−0.35 ± 0.04^ABa^
Germination percentage	0.05	−0.16 ± 0.01^Aa^	−0.23 ± 0.06^Aa^	−0.19 ± 0.07^Aa^	−0.23 ± 0.04^Aa^
	0.1	−0.32 ± 0.01^Bb^	−0.22 ± 0.00^Aa^	−0.25 ± 0.01^ABab^	−0.32 ± 0.04^Bab^
	0.2	−0.15 ± 0.03^Aa^	−0.18 ± 0.00^Aa^	−0.34 ± 0.02^Bbc^	−0.34 ± 0.00^Bb^
	0.5	−0.36 ± 0.03^Ab^	−0.34 ± 0.02^Ab^	−0.44 ± 0.01^Bc^	−0.38 ± 0.00^ABb^
Shoot length	0.05	−0.02 ± 0.04^Aa^	−0.15 ± 0.03^Bb^	−0.06 ± 0.02^Aa^	−0.22 ± 0.01^Bc^
	0.1	0.09 ± 0.02^Bab^	−0.02 ± 0.01^Aa^	−0.12 ± 0.01^BCb^	−0.14 ± 0.01^Cb^
	0.2	−0.21 ± 0.02^Bb^	−0.09 ± 0.03^Ab^	−0.14 ± 0.02^Ab^	−0.06 ± 0.02^Aa^
	0.5	−0.13 ± 0.03^Abc^	−0.17 ± 0.02^ABb^	−0.21 ± 0.02^Bc^	−0.20 ± 0.01^ABc^
Root length	0.05	−0.34 ± 0.03^Aa^	−0.41 ± 0.02^Aa^	−0.39 ± 0.04^Aa^	−0.39 ± 0.03^Aa^
	0.1	−0.40 ± 0.03^Aab^	−0.41 ± 0.03^Aa^	−0.50 ± 0.03^Aa^	−0.42 ± 0.06^Aab^
	0.2	−0.43 ± 0.01^Ab^	−0.46 ± 0.02^ABa^	−0.50 ± 0.01^Ba^	−0.50 ± 0.02^Bab^
	0.5	−0.53 ± 0.02^Ac^	−0.49 ± 0.04^Aa^	−0.50 ± 0.04^Aa^	−0.54 ± 0.02^Ab^

### Antioxidant enzyme activity in aboveground part and roots of seedlings

For the aboveground part, the extract concentrations had significant (*df* = 4; *P* < 0.001) effect on the SOD activity, and both the extract concentrations and the years of planting had significant (*df* = 4; *P* < 0.001) effect on the POD activity. Their interactions on the SOD and POD activities were significant (*df* = 12; *P* < 0.001), but there was no clear pattern for the activity in response to the extract concentrations and the years of planting (Table 3). For *E. sibiricus* planted for 5 years, the SOD activity was significantly lower than the control. At 0.1 g/mL of the extract from *E. sibiricus* planted for 8 years, the POD activity was significantly higher than that of the control. The POD activity firstly increased and then decreased with the increase of planting years at the extract concentrations from 0.2 to 0.5 g/mL.

**Table 3:** Effects of water extract of rhizosphere soils of *E. sibiricus* in different planting years on the superoxide dismutase (SOD) and peroxidase (POD) activity in aboveground seedlings of *E. sibiricus*. Means with different superscript lowercase letters indicate significant differences between different concentrations of the same planting years, and upper case letters indicate significant differences between different planting years of the same concentration at *P* < 0.05 (LSD test).

	Concentrations of soil extract (g/mL)	Years of planting				Significance (*P* values)		
		3	4	5	8	Years	Concentrations	Y ×C
SOD (U/g)	0	197.13 ± 3.11^Aa^	197.13 ± 3.11^Aa^	197.13 ± 3.11^Aa^	197.13 ± 3.11^Aa^	0.00	0.00	0.00
	0.05	198.16 ± 5.24^Aa^	204.23 ± 3.32^Aa^	153.39 ± 2.14^Cc^	169.95 ± 1.63^Bc^			
	0.1	183.85 ± 7.44^Aa^	167.86 ± 1.32^Ab^	179.83 ± 3.59^Ab^	184.16 ± 4.99^Ab^			
	0.2	188.97 ± 7.99^Aa^	160.26 ± 1.04^Bb^	163.71 ± 0.42^Bc^	182.17 ± 3.01^Ab^			
	0.5	160.53 ± 2.95^Bb^	203.7 ± 11.13^Aa^	160.76 ± 4.77^Bc^	199.97 ± 1.61^Aa^			
POD (U/mg. min)	0	33.37 ± 1.35^Aa^	33.37 ± 1.35^Aa^	33.37 ± 1.35^Aa^	33.37 ± 1.35^Ab^	0.00	0.00	0.00
	0.05	24.10 ± 0.96^Bb^	29.88 ± 1.29^Aa^	23.90 ± 1.17^Bb^	20.77 ± 1.28^Bd^			
	0.1	39.40 ± 4.51^Aa^	25.55 ± 0.82^Bb^	38.69 ± 2.78^Aa^	43.80 ± 1.69^Aa^			
	0.2	23.81 ± 3.69^Cb^	31.68 ± 0.77^ABa^	34.12 ± 1.41^Aa^	25.23 ± 1.42^BCc^			
	0.5	23.67 ± 0.74^Bb^	25.19 ± 1.56^Bb^	36.49 ± 1.28^Aa^	24.3 ± 0.81^Bcd^			

As for the roots of *E. sibiricus* seedlings, the planting years, extract concentrations, and their interactions significantly affected the SOD and POD activities (Table 4). For the extract from *E. sibiricus* planted for 5 years, the SOD and POD activities firstly increased and then decreased with the increase of the extract concentrations, and the SOD and POD activities were significantly higher than those for the other treatments at the extract concentrations of 0.1 and 0.2 g/mL. There appeared a quadratic pattern between the SOD activity and the planting years at 0.1 g/mL of the extract concentration, and between the POD activity and the planting years at 0.2 g/mL of the the extract concentration.

**Table 4:** Effects of water extract of rhizosphere soils of *E. sibiricus***in different planting years on the superoxide dismutase (SOD) and peroxidase (POD) activity in***E. sibiricus* roots. Means with different superscript lowercase letters indicate significant differences between different concentrations of the same planting years, and upper case letters indicate significant differences between different planting years of the same concentration at *P* < 0.05 (LSD test).

	Concentrations of soil extract (g/mL)	Years of planting				Significance (*P* values)		
		3	4	5	8	Years	Concentrations	Y ×C
SOD (U/g)	0	123.71 ± 4.30^Abc^	123.71 ± 4.30^Ab^	123.71 ± 4.30^Ac^	123.71 ± 4.30^Abc^	0.00	0.00	0.00
	0.05	116.71 ± 1.01^Ac^	115.27 ± 3.83^Ab^	145.65 ± 2.57^Ab^	147.21 ± 14.82^Aab^			
	0.1	134.43 ± 6.66^Bb^	165.69 ± 2.85^ABa^	180.72 ± 2.72^Aa^	98.14 ± 7.58^Cc^			
	0.2	151.38 ± 4.53^Ba^	101.57 ± 1.34^Cc^	112.29 ± 5.14^Cd^	184.01 ± 2.34^Aa^			
	0.5	136.63 ± 2.56^Ab^	101.19 ± 1.10^Bc^	76.07 ± 1.14^Ce^	141.35 ± 4.16^Aabc^			
POD (U/mg. min)	0	34.86 ± 0.68^Ad^	34.86 ± 0.68^Ac^	34.86 ± 0.68^Ac^	34.86 ± 0.68^Ac^	0.00	0.00	0.00
	0.05	46.84 ± 3.05^Cc^	25.00 ± 1.70^Dd^	78.38 ± 2.55^Ab^	61.90 ± 0.92^Bb^			
	0.1	79.99 ± 5.17^Aa^	73.92 ± 2.83^ABa^	82.03 ± 1.29^Ab^	63.50 ± 2.39^Bb^			
	0.2	57.27 ± 1.12^Cb^	70.52 ± 1.08^Ba^	106.02 ± 2.61^Aa^	59.51 ± 1.69^Cb^			
	0.5	81.49 ± 0.95^Aa^	52.23 ± 1.79^Cb^	78.90 ± 2.43^Ab^	70.19 ± 2.66^Ba^			

### Concentrations of free proline, soluble sugar, soluble protein and MDA in aboveground part and roots of seedlings

For the aboveground part, the planting years, extract concentrations, and their interactions significantly affected these concentrations (Table 5). Compared with that for the control, the free proline concentration was decreased substantially by the soil extract, and the proline concentration declined with the increase of the planting years at extract concentrations of 0.05 g/mL. The soluble sugar concentration increased with the planting years under the soil extract treatments, except for 3 planting years where the sugar concentration was decreased by the soil extract compared with that for the control. The sugar concentration was particularly higher for the extract concentrations of 0.2 g/mL for 4 and 5 planting years. The soluble protein concentration was, in general, lowered by the soil extract compared with the control, depending on its extract concentrations and the planting years, and particularly lower for the extract concentrations of 0.05 g/mL for 4 and 5 years of planting and 0.2 g/mL for 3 years of planting. Compared with the control, the MDA concentration was, in general, increased by the soil extract, except for at the 0.05 g/mL concentration for 4 planting years which was actually lower (*P* = 0.00); the MDA concentration was particularly higher at the soil extract concentrations of 0.1 g/mL for 3 planting years, 0.2 g/mL for 4 planting years, 0.1 g/mL for 5 planting years, and 0.5 g/mL for 8 planting years.

**Table 5:** Effects of water extract of rhizosphere soils of *E. sibiricus* in different planting years on the concentrations of free proline, soluble sugar, soluble protein, and malondialdehyde (MDA) in aboveground seedlings of *E. sibiricus*. Means with different superscript lowercase letters indicate significant differences between different concentrations of the same planting years, and upper case letters indicate significant differences between different planting years of the same concentration at *P* < 0.05 (LSD test).

	Concentrations of soil extract (g/mL)	Years of planting				Significance (*P* values)		
		3	4	5	8	Years	Concentrations	Y ×C
Free proline (µg/g)	0	71.26 ± 1.88^Aa^	71.26 ± 1.88^Aa^	71.26 ± 1.88^Aa^	71.26 ± 1.88^Aa^	0.00	0.00	0.00
	0.05	17.17 ± 0.56^Ad^	15.80 ± 0.86^Ac^	14.20 ± 1.36^Ac^	13.95 ± 1.41^Ab^			
	0.1	37.86 ± 1.89^Ab^	24.55 ± 0.92^Bb^	24.28 ± 1.66^Bb^	16.89 ± 1.31^Cb^			
	0.2	37.93 ± 1.11^Ab^	18.93 ± 0.88^Cc^	23.59 ± 1.81^Bb^	16.60 ± 0.73^Cb^			
	0.5	31.12 ± 1.01^Ac^	18.38 ± 0.65^Bc^	14.30 ± 0.96^Cc^	17.99 ± 1.58^Bb^			
Soluble sugar (mg/g)	0	19.24 ± 0.96^Aa^	19.24 ± 0.96^Ac^	19.24 ± 0.96^Ac^	19.24 ± 0.96^Ab^	0.00	0.00	0.00
	0.05	8.84 ± 0.20^Bb^	18.93 ± 0.67^Ac^	17.87 ± 1.19^Ac^	17.23 ± 1.52^Ab^			
	0.1	4.83 ± 0.32^Cc^	19.25 ± 1.25^Bc^	24.46 ± 1.70^Ab^	17.94 ± 1.23^Bb^			
	0.2	7.49 ± 0.23^Db^	26.96 ± 0.57^Ba^	31.03 ± 0.93^Aa^	23.28 ± 0.14^Ca^			
	0.5	4.82 ± 0.25^Cc^	22.66 ± 0.43^Bb^	27.11 ± 1.18^Ab^	23.88 ± 1.00^Ba^			
Soluble protein (mg/g)	0	5.82 ± 0.06^Aa^	5.82 ± 0.06^Aa^	5.82 ± 0.06^Ab^	5.82 ± 0.06^Ab^	0.00	0.00	0.00
	0.05	5.33 ± 0.13^Ab^	5.06 ± 0.02^Bc^	4.76 ± 0.06^Cc^	4.70 ± 0.01^Cc^			
	0.1	5.56 ± 0.07^BCab^	5.31 ± 0.12^Cb^	6.02 ± 0.05^Ab^	5.69 ± 0.06^Bb^			
	0.2	4.79 ± 0.06^Cc^	5.32 ± 0.03^Bb^	5.99 ± 0.18^Ab^	6.27 ± 0.07^Aa^			
	0.5	5.54 ± 0.09^Cab^	5.72 ± 0.04^BCa^	7.12 ± 0.14^Aa^	5.87 ± 0.06^BCb^			
MDA (µmol/g)	0	15.64 ± 1.30^Ac^	15.64 ± 1.30^Ab^	15.64 ± 1.30^Ac^	15.64 ± 1.30^Ad^	0.00	0.00	0.00
	0.05	24.06 ± 0.36^Ab^	11.15 ± 0.65^Dc^	22.47 ± 0.04^Bb^	20.81 ± 0.09^Cc^			
	0.1	29.78 ± 2.53^Aa^	16.28 ± 0.10^Cb^	27.37 ± 0.54^Aa^	22.46 ± 0.17^Bc^			
	0.2	17.22 ± 0.94^Cc^	25.20 ± 0.27^Ba^	24.09 ± 0.30^Bb^	27.29 ± 0.23^Ab^			
	0.5	19.08 ± 0.30^Cc^	23.09 ± 1.02^Ba^	22.79 ± 0.09^Bb^	32.45 ± 0.90^Aa^			

**Table 6:** Effects of water extract of rhizosphere soils of *E. sibiricus* in different planting years on the concentrations of free proline, soluble sugar, soluble protein, and malondialdehyde (MDA) in *E. sibiricus* roots. Means with different superscript lowercase letters indicate significant differences between different concentrations of the same planting years, and upper case letters indicate significant differences between different planting years of the same concentration at *P* < 0.05 (LSD test).

	Concentrations of soil extract (g/mL)	Years of planting				Significance (*P* values)		
		3	4	5	8	Years	Concentrations	Y ×C
Free proline (µg/g)	0	23.64 ± 0.89^Aa^	23.64 ± 0.89^Aa^	23.64 ± 0.89^Ad^	23.64 ± 0.89^Ab^	0.00	0.00	0.00
	0.05	8.57 ± 1.07^Dc^	12.22 ± 0.90^Cb^	29.95 ± 0.8^8Ac^	15.79 ± 0.61^Bc^			
	0.1	8.14 ± 0.61^Cc^	25.23 ± 1.46^Ba^	31.75 ± 1.09^Ac^	22.76 ± 0.18^Bb^			
	0.2	13.88 ± 0.60^Cb^	14.74 ± 0.69^Cb^	53.20 ± 1.00^Aa^	25.53 ± 0.74^Bb^			
	0.5	20.99 ± 1.15^Ba^	8.51 ± 1.01^Cc^	35.32 ± 0.81^Ab^	36.92 ± 1.71^Aa^			
Soluble sugar (mg/g)	0	15.60 ± 0.37^Aa^	15.60 ± 0.37^Ab^	15.60 ± 0.37^Ac^	15.60 ± 0.37^Aa^	0.00	0.00	0.00
	0.05	7.94 ± 0.89^Cc^	9.77 ± 0.02^Cc^	14.03 ± 0.41^Bc^	16.71 ± 0.89^Aa^			
	0.1	10.86 ± 0.94^Bb^	9.99 ± 0.11^Cc^	14.23 ± 0.75^Ac^	16.01 ± 0.91^Aa^			
	0.2	17.73 ± 0.64^Aa^	17.74 ± 0.10^Aa^	18.37 ± 0.88^Ab^	15.66 ± 1.64^Aa^			
	0.5	15.77 ± 0.29^Ba^	16.22 ± 0.25^Bb^	25.40 ± 0.94^Aa^	10.74 ± 0.28^Cb^			
Soluble protein (mg/g)	0	5.18 ± 0.02^Aab^	5.18 ± 0.02^Abc^	5.18 ± 0.02^Ab^	5.18 ± 0.02^Ac^	0.02	0.00	0.00
	0.05	4.50 ± 0.37^Bb^	5.37 ± 0.14^Ab^	3.58 ± 0.01Cd	3.59 ± 0.03^Cd^			
	0.1	5.40 ± 0.23^Aa^	4.68 ± 0.10^Bc^	4.86 ± 0.02^Bc^	5.15 ± 0.18^ABc^			
	0.2	5.25 ± 0.14^Aab^	5.11 ± 0.36^Abc^	5.71 ± 0.04^Aa^	5.50 ± 0.08^Ab^			
	0.5	4.95 ± 0.22^Bab^	5.88 ± 0.06^Aa^	5.26 ± 0.11^Bb^	6.21 ± 0.05^Aa^			
MDA (µmol/g)	0	4.45 ± 0.63^Ad^	4.45 ± 0.63^Ac^	4.45 ± 0.63^Ac^	4.45 ± 0.63^Ae^	0.00	0.00	0.00
	0.05	5.41 ± 0.17^Cd^	8.16 ± 0.43^Ab^	7.08 ± 0.10^Bb^	6.32 ± 0.20^Bd^			
	0.1	9.05 ± 0.26^Bc^	8.27 ± 0.22^Bb^	8.45 ± 0.27^Bb^	12.17 ± 0.21^Ab^			
	0.2	10.95 ± 0.17^Bb^	11.20 ± 1.94^Bab^	12.11 ± 0.83^Ba^	21.92 ± 0.05^Aa^			
	0.5	17.94 ± 0.06^Aa^	13.52 ± 1.48^Ba^	12.53 ± 0.52^Ba^	8.29 ± 0.11^Cc^			

For the roots of *E. sibiricus* seedlings, the soil extract concentrations, planting years, and their interactions had significant (*P* < 0.001) effect on the concentrations of free proline, soluble sugar, soluble protein, and MDA (Table 6). Compared with the control, the proline concentration was lowered by the soil extract for 3 and 4 planting years, but increased for 5 and 8 planting years, depending on the extract concentration. The proline concentration was particularly higher at 0.2 g/mL of the soil extract for 5 planting years compared with the control (*P* < 0.001). The soluble sugar concentration was decreased by the soil extract at 0.05 and 0.1 g/mL concentrations for 3 and 4 planting years and 0.5 g/mL for 8 planting years. The soluble protein concentration was decreased by the soil extract at 0.05 g/mL for 5 and 8 planting years, but increased by the soil extract at 0.2 g/mL for 5 planting years and 0.2 and 0.5 g/mL for 8 planting years. The MDA concentration was increased by the soil extract, in general, in a dose-dependent manner, and significantly higher than that of the control.

### Phytohormone concentrations in aboveground part and roots of seedlings

The soil extract concentrations, planting years, and their interactions had significant influences on these concentrations (*P* < 0.001) (Table 7). For the aboveground part, compared with that for the control, the IAA and GA concentrations were lowered by the soil extract regardless the planting years. The IAA concentration increased linearly with the soil extract concentrations for 3 planting years and changed quadratically with the soil extract concentrations for 4, 5, and 8 planting years. The GA concentration declined linearly with the soil extract concentrations for 3, 4, and 8 planting years, but increased linearly with the soil extract concentrations for 5 planting years. The ABA concentration varied substantially with the soil extract concentrations in conjunction with the planting years, it was particularly high at 0.50 g/mL of the soil extract concentration for 4 planting years, but low at 0.05 g/mL of the soil extract concentration for 3 planting years, 0.05 and 0.1 g/mL of the extraction concentrations for 5 planting years, and 0.5 g/mL of the extract concentration for 8 planting years.

**Table 7:** Effects of water extract of rhizosphere soils of ***E. sibiricus*in different planting years on the concentrations of indole acetic acid (IAA), gibberellin (GA), and abscisic acid (ABA) in aboveground seedlingsof***E. sibiricus*. Means with different superscript lowercase letters indicate significant differences between different concentrations of the same planting years, and upper case letters indicate significant differences between different planting years of the same concentration at *P* < 0.05 (LSD test).

	Concentrations of soil extract (g/mL)	Years of planting				Significance (*P* values)		
		3	4	5	8	Years	Concentrations	Y ×C
IAA (µg/g)	0	1.86 ± 0.01^Aa^	1.86 ± 0.01^Aa^	1.86 ± 0.01^Aa^	1.86 ± 0.01^Aa^	0.00	0.00	0.00
	0.05	1.03 ± 0.01^Ae^	0.79 ± 0.08^Bc^	0.92 ± 0.06^ABc^	1.04 ± 0.02^Ad^			
	0.1	1.15 ± 0.02^Bd^	0.85 ± 0.02^Cc^	1.11 ± 0.08^Bb^	1.30 ± 0.02^Ac^			
	0.2	1.34 ± 0.04^BCc^	1.43 ± 0.02^ABb^	1.27 ± 0.06^Cb^	1.52 ± 0.04^Ab^			
	0.5	1.70 ± 0.01^Ab^	0.85 ± 0.05^Cc^	1.11 ± 0.02^Bb^	1.04 ± 0.04^Bd^			
GA (µg/g)	0	39.17 ± 1.02^Aa^	39.17 ± 1.02^Aa^	39.17 ± 1.02^Aa^	39.17 ± 1.02^Aa^	0.00	0.00	0.00
	0.05	25.30 ± 0.74^Ab^	24.00 ± 0.66^ABb^	14.82 ± 0.70^Cd^	22.26 ± 0.77^Bb^			
	0.1	25.75 ± 1.13^Ab^	21.64 ± 0.85^Bbc^	20.07 ± 0.63^Bc^	19.18 ± 0.51^Bc^			
	0.2	24.49 ± 0.07^Abc^	20.87 ± 0.35^Cc^	22.85 ± 0.04^Bb^	18.49 ± 0.31^Dc^			
	0.5	21.92 ± 1.17^Ac^	16.4 ± 0.74^Bd^	24.00 ± 0.01^Ab^	17.51 ± 0.89^Bc^			
ABA (ng/g)	0	131.93 ± 1.01^Aa^	131.93 ± 1.01^Abc^	131.93 ± 1.01^Aab^	131.93 ± 1.01^Ac^	0.00	0.00	0.00
	0.05	52.22 ± 5.74^Cd^	113.20 ± 11.68^Bc^	63.90 ± 4.78^Cd^	172.86 ± 3.41^Aa^			
	0.1	92.72 ± 13.53^Bbc^	128.10 ± 2.08^Abc^	84.65 ± 12.79^Bc^	145.33 ± 0.10^Ab^			
	0.2	119.43 ± 11.58^Bab^	154.60 ± 8.23^Ab^	150.58 ± 0.85^Aa^	70.66 ± 4.11^Cd^			
	0.5	85.27 ± 6.92^Cc^	349.74 ± 12.7^Aa^	128.06 ± 1.77^Bb^	57.42 ± 5.38^De^			

For the roots of *E. sibiricus* seedlings, the IAA concentration was increased by the soil extract compared with that for the control, except for the extract concentrations of 0.2–0.5 g/mL for 4 planting years, 0.1 mg/mL for 5 planting years, and 0.5 g/mL for 8 planting years (Table 8). Compared with that for the control, the GA concentration was higher at the soil extract concentrations of 0.05 g/mL for 4 (*P* = 0.00) and 5 planting years (*P* < 0.001), but lower at the other extract concentrations except for the soil extract concentrations of 0.1 g/mL for 3 planting years and 0.2 g/mL for 8 planting years. The ABA concentration was increased by the soil extract at the concentrations of 0.05, 0.1, and 0.2 g/mL for 3 planting years, 0.1 and 0.5 g/mL for 4 planting yeart, 0.05 and 0.1 g/mL for 5 planting years, and 0.5 g/mL for 8 planting years.

**Table 8:** Effects of water extract of rhizosphere soils of *E. sibiricus* in different planting years on the concentrations of indole acetic acid (IAA), gibberellin (GA), and abscisic acid (ABA) in *E. sibiricus* roots. Effects of water extra Means with different superscript lowercase letters indicate significant differences between different concentrations of the same planting years, and upper case letters indicate significant differences between different planting years of the same concentration at *P* < 0.05 (LSD test).

	Concentrations of soil extract (g/mL)	Years of planting				Significance (*P* values)		
		3	4	5	8	Years	Concentrations	Y ×C
IAA (µg/g)	0	0.02 ± 0.00^Ad^	0.02 ± 0.00^Ac^	0.02 ± 0.00^Ac^	0.02 ± 0.00^Ac^	0.00	0.00	0.00
	0.05	0.07 ± 0.00^Bb^	0.16 ± 0.00^Aa^	0.05 ± 0.00^Cb^	0.04 ± 0.01^Cb^			
	0.1	0.27 ± 0.00^Aa^	0.11 ± 0.01^Bb^	0.02 ± 0.00^Dc^	0.04 ± 0.00^Cb^			
	0.2	0.03 ± 0.00^Cc^	0.01 ± 0.00^Dc^	0.08 ± 0.00^Ba^	0.13 ± 0.00^Aa^			
	0.5	0.03 ± 0.00^Cc^	0.00 ± 0.00^Bc^	0.04 ± 0.01^Ab^	0.01 ± 0.00^Ac^			
GA (µg/g)	0	4.08 ± 0.05^Aa^	4.08 ± 0.05^Ab^	4.08 ± 0.05^Ab^	4.08 ± 0.05^Aa^	0.00	0.00	0.00
	0.05	3.86 ± 0.04^Cb^	4.25 ± 0.06^Ba^	4.71 ± 0.06^Aa^	2.82 ± 0.16^Dc^			
	0.1	4.15 ± 0.09^Aa^	3.68 ± 0.00^Bc^	3.24 ± 0.01^Cc^	3.25 ± 0.11^Cb^			
	0.2	3.66 ± 0.00^Ac^	3.34 ± 0.00^Bd^	2.65 ± 0.03^Cd^	3.88 ± 0.15^Aa^			
	0.5	3.37 ± 0.01^Bd^	3.62 ± 0.01^Ac^	2.51 ± 0.07^Dd^	2.99 ± 0.07^Cbc^			
ABA (ng/g)	0	3.67 ± 0.06^Ad^	3.67 ± 0.06^Ac^	3.67 ± 0.06^Ac^	3.67 ± 0.06^Ab^	0.00	0.00	0.00
	0.05	14.46 ± 0.24^Ba^	4.40 ± 0.26^Bc^	52.07 ± 4.13^Aa^	8.49 ± 1.09^BCb^			
	0.1	11.24 ± 0.12^Bb^	7.76 ± 0.69^BCb^	24.55 ± 4.01^Ab^	4.23 ± 0.08^Cb^			
	0.2	9.78 ± 0.11^Ac^	4.16 ± 0.02^Bc^	9.75 ± 1.46^Ac^	5.84 ± 0.55^Bb^			
	0.5	4.06 ± 0.01^Bd^	9.81 ± 1.08^Ba^	8.47 ± 1.49^Bc^	22.65 ± 4.23^Aa^			

## Discussion

Plants usually release allelochemicals to the rhizosphere under field conditions (Zhang et al., 2009) and long-term continuous cropping the same species could result in accumulation of autotoxic substances, causing autotoxicity on plants (Cheng et al., 2020). For example, *Lolium rigidum* and *Medicago sativa* have been proved to release allelochemical compounds (Canals, Emeterio & Peralta, 2005; Li et al., 2020). In the present study, we showed that the water extract of rhizosphere soils from *E. sibiricus* plants inhibited the seeds germination and growth of seedlings (both the aboveground part and roots) of *E. sibiricus*, and the inhibitory effects appeared to be stronger with the higher concentration of the extract and the longer years of planting. The results suggest that *E. sibiricus* also has such an autotoxic phenomenon and the rhizosphere soils contain autotoxic substances, which helps to explain the rapid decline of grassland productivity of *E. sibiricus* after 2–3 years of artificial grassland planting (Quan et al., 2021). In addition, the inhibitory effect on the root length of *E. sibiricus* was more profound than that on the shoot length in this study, which is agreed with the proposal that different parts of *E. sibiricus* have different responses to autotoxicity (Zhang et al., 2021b). Sun & He (2019) found that soil phosphorus availability mediate the autotoxicity of plant root exudation, and the addition of activated carbon and water-soluble phosphorus fertilizers increased the biomass of *Lactuca sativa*, this result guides that we can try to add activated carbon and phosphorus availability to relieve the autotoxicity during the decline period of the *E. sibiricus* artificial grassland.

Allelochemicals could prompt accumulation of ROS in plant seedlings. If superfluous ROS cannot be scavenged in time, it will cause apoptosis (Xin et al., 2019). Antioxidant enzymes play critical roles in maintaining the intracellular redox homeostasis (Gill & Tuteja, 2010). In the present study, the SOD and POD activities in *E. sibiricus* seedlings were changed by autotoxicity. The SOD activity was lowered or unchanged in the aboveground part of *E. sibiricus* seedlings, whereas the activity was enhanced (at 0.1−0.2 g/mL of the soil extract concentrations) or lowered in the roots, depending on the soil extract concentration and the years of planting, Zeng et al. (2022) discovered that SOD and POD activities in plants were reduced by abiotic stress. The results in the current study suggest that the capacity of partitioning superoxide radicals may be reduced in the aboveground part, but may be increased in the roots at the particular concentration of the soil extract. As for the POD activity, it was increased or decreased in the aboveground part, enhanced in the roots by the soil extract, the magnitude of the change varied for the differences in the soil extract concentrations and the years of planting. It is difficult to explain such inconsistent results in *E. sibiricus*. In tomato plants, there was no significant change in the metabolism of ROS by autotoxin (Soltys et al., 2012). The ROS mechanisms of autotoxicity seem to be highly complex.

One of the results of oxidative stress is damage of the cell membranes, leading to the disruption of intracellular homeostasis and affecting plant growth and development (Huang et al., 2013; Jespersen, Yu & Huang, 2017). Osmotic substances, including proline, soluble sugar, and soluble protein, can maintain the cell turgor so that stabilize the membrane system (Fang & Xiong, 2015). Free proline can be accumulated in plants under a stress to regulate osmotic pressure, maintain cell health and resist adverse conditions (Sun et al., 2016). Soluble sugar and soluble protein are positively correlated with the stress degree of plants, and abiotic stress can significantly increased soluble sugar and soluble protein contents in plants (Zhang et al., 2019). In the present study, the free proline concentration in both the aboveground part and roots of *E. sibiricus* seedlings was reduced by the soil extract, particularly in the aboveground part, and the reduction appeared to become severe with the duration of planting years for the aboveground part, but was lesser in the roots. So, the ability of resisting stress to autotoxicity of *E. sibiricus* was reduced, as there is a positive correlation between the free proline content and the plant survival rate (Jankovska-Bortkevič et al., 2019). The soluble sugar concentration was increased by the soil extract in the aboveground part of the seedlings except for those from 3 planting years, but in the roots, the changes in the sugar concentration varied without a clear pattern in this study. The results may suggest that the seedling roots were damaged by allelochemicals in rhizosphere soils and the synthesis of soluble sugar in the roots was disrupted. Our results are supported by a previous reports that autotoxicity could cause abnormal root functions (Zhang et al., 2020b). When autotoxicity produced by plants stimulates the plasma membranes, which induce plants to produce metabolites that damage cell membranes (Yang et al., 2018).

The MDA concentration can be used to indicate stability of the cell membranes under stress (Yu et al., 2009; Yang et al., 2011; Lan et al., 2017). In the present study, the MDA concentration in both the aboveground part and roots of *E. sibiricus* seedlings was increased by the soil extract, in general, the increase was more substantial in the roots than those in the aboveground part, as well as with the higher concentrations of the soil extract. The results may indicate that there is coordination in the whole plant of *E. sibiricus* seedlings when resisting autotoxic stress, and the high concentration of the soil extract causes more severe damage to the cell membranes. Wang et al. (2021) discovered that continuous accumulation of autotoxic substances in rhizosphere soils with the increase of planting years was the key factor that caused the obstacle of alfalfa continuous cropping.

The interactions among, and regulation of, plant endogenous hormones are important in acclimation to adverse environments (Anwar et al., 2018). Phytohormones, such as IAA, GA, and ABA, are essential substances for plant growth, development and improving stress resistance, and they regulate the defense system and improve the tolerance of plants to different stresses (Krishnamurthy & Rathinasabapathi, 2013; Li et al., 2018). In the present study, the IAA concentration was lowered by the soil extract in the aboveground part, but varied in the roots of the seedlings; The GA concentration was lowered by the soil extract in both the aboveground part and roots of seedlings; The ABA concentration varied in the aboveground part and tuned out to increase in the roots. The results suggest that the autotoxicity of *E. sibiricus* may reduce the growth promoting hormone in plants, leading to the unbalanced synthesis of plant hormones. The results corroborate those of other studies, in which allelopathy changed the biosynthesis of plant hormones (Zhang, Wang & Li, 2021a). There are differences in plant hormone synthesis in plant roots and seedlings under the same stress conditions (Bai et al., 2010). During the growth of *E. sibiricus* grassland, autotoxicity causes the decline of *E. sibiricus* grassland with the increase of years, which may be caused by the autotoxic substances in the rhizosphere soils affecting the physiological function and hormone synthesis of *E. sibiricus*, and the results of the study provide the theoretical basis for the management of *E. sibiricus* artificial grassland.

## Conclusion

The results in this study showed the inhibitory effects of the rhizosphere soil extract of *E. sibiricus* on seed germination and seedling growth of *E. sibiricus*, supporting our hypothesis that autotoxicity can be a contributor to the retardation of the growth and development of artificial *E. sibiricus* grasslands. The inhibitory effects could be attributed to impaired antioxidant capacity, and disturbance of osmotic-regulatory substances and plant hormones, and were more profound on the roots than on the aboveground part of the seedlings. The present study demonstrated that autotoxicity may be one of the mechanisms causing decline of the *E. sibiricus*. Therefore, it is necessary to further investigate the autotoxic substances content in rhizosphere soils of *E. sibiricus* and explore methods to alleviate autotoxicity.

## Supplemental Information

10.7717/peerj.13768/supp-1 Supplemental Information 1 Effects of water extract of rhizosphere soils of *Elymus sibiricus* in different planting years on seeds germination, seedlings growth, physiological characteristics and plant hormones Each data indicates the effects of different years and concentrations of rhizosphere soil extracts of *Elymus sibiricus* on the studied indexes.

## Additional Information and Declarations

### Data Availability

The following information was supplied regarding data availability: The raw data are available in the Supplementary File.
